# Assessing the joint effect of population stratification and sample selection in studies of gene-gene (environment) interactions

**DOI:** 10.1186/1471-2156-13-5

**Published:** 2012-01-27

**Authors:** KF Cheng, JY Lee

**Affiliations:** 1 Biostatistics Center and Graduate Institute of Biostatistics, China Medical University, Taichung, Taiwan; 2 Graduate Institute of Statistics, National Central University, Chungli, Taiwan

## Abstract

**Background:**

It is well known that the presence of population stratification (PS) may cause the usual test in case-control studies to produce spurious gene-disease associations. However, the impact of the PS and sample selection (SS) is less known. In this paper, we provide a systematic study of the joint effect of PS and SS under a more general risk model containing genetic and environmental factors. We provide simulation results to show the magnitude of the bias and its impact on type I error rate of the usual chi-square test under a wide range of PS level and selection bias.

**Results:**

The biases to the estimation of main and interaction effect are quantified and then their bounds derived. The estimated bounds can be used to compute conservative p-values for the association test. If the conservative p-value is smaller than the significance level, we can safely claim that the association test is significant regardless of the presence of PS or not, or if there is any selection bias. We also identify conditions for the null bias. The bias depends on the allele frequencies, exposure rates, gene-environment odds ratios and disease risks across subpopulations and the sampling of the cases and controls.

**Conclusion:**

Our results show that the bias cannot be ignored even the case and control data were matched in ethnicity. A real example is given to illustrate application of the conservative p-value. These results are useful to the genetic association studies of main and interaction effects.

## Background

In the search of causative agents of human disease, both environmental and genetic risk factors have been identified. Overwhelming evidence indicates that there are reasons to believe that relative common polymorphisms in a wide spectrum of genes may modify the effect of environmental agents [1,2]. Several studies also have demonstrated the presence of gene-gene interaction in complex human diseases [3-7]. Gene-gene interaction, or epistasis, is also considered as a basic genetic concept which has been widely used by biologists for a long time [8].

Many association designs have been proposed for studying gene-environment or gene-gene interactions. Recently, Wang and Zhao [9] found that in the study of gene-gene interactions, the unmatched case-control association design is more powerful than both the matched case-control design and case-parents design. They also found that when a logistic regression model is fitted for assessing gene-environment interactions based on case-parents sample, the approach may be susceptible to the PS bias [10]. However, case-control design is also well known to be susceptible to the PS bias in the study of genetic effect, if the gene under study shows marked variation in allele frequency across subgroups of the population and if these subgroups also differ in their base-line disease risks [11-17]. Wang, et al. [18] recently provided numerical examples showing that when the correlation between genetic and environmental factors is small or the linkage disequilibrium is weak, and case-control data were collected according to a simple random sampling (SRS) scheme, that is no selection bias, the PS bias in testing null interaction odds ratio is also small. However, selection bias often occurs in case-control studies and more studies are needed in order to better understand the impact of the PS and SS.

In this paper, we investigate the joint effect of population stratification and sample selection in testing null main or interaction effects. Under general sampling, we quantify the magnitude of the PS-SS bias in terms of the baseline disease risks, genotype frequencies, exposure rates, their odds ratios (linkage disequilibrium coefficients), and the effect sizes of the risk factors. Based on this result, we find that matching in ethnicity cannot eliminate bias in association studies. Using the bias, we are also able to derive important conditions under which it is null.

The PS-SS bias cannot be estimated, since we don't know how many subpopulations involved in the studied population and/or which subpopulation a person belongs to. Although adjusting for covariates such as principal components can be used to account for PS in genome wide association studies [19], however, it is not clear whether the same approach can be applied in the studies of interaction. Since, for example, the bias level also depends on the effect size of the environmental factor. In this paper, we also derive useful bounds to measure the maximal impact of the bias. Sometimes, these bounds can be estimated so that tests robust to the joint effect of PS and SS can be derived; see Lee and Wang [20] for similar suggestion in studies of gene-disease association. We use theoretical formula and simulation results to show the general properties of the usual association test in the presence of PS or selection bias. We also provide a real example to demonstrate computation of a conservative p-value in studying interaction effect of maternal smoking and GSTT1 variant on the risk of orofacial cleft.

## Results

### The Magnitude of the Bias

We begin this section with the notation that will be used throughout this work. Disease status is denoted as *D *with levels *D *= 1, and 0, indicating the presence and absence of the disease, respectively. Let *G *= 1(0) represent the presence (absence) of the genotype of interest. *H *= 1(0) represents the presence (absence) of the environmental exposure or another genotype of interest. Although we only focus on 2 × 2 × 2 table, however, all results can be extended to any number of risk factors or any number of levels. We also assume that the population under study consists of *K *subpopulations and denote *S *as the stratification variable, taking values *s *= 1,..., *K*. However, *K *is unknown and *S *is not observable in our discussion of the PS effect.

To quantify the PS effect, we assume that the risk model is given by

$$\begin{array}{ccc} \text{logit}\;P(D= & 1|G=g,H=h,S=s) & \\ & =\mu^{\prime }+{\alpha^{\prime }}_{s}+\beta g+\gamma h+\delta gh, \end{array}$$

where the genetic and environmental data are obtained from subpopulation *s*. As usual, we use *s *= 1, *g *= 0, and *h *= 0 to represent the referent subpopulation, genotype and environmental exposure, respectively. For the purpose of identifiability, we define $\alpha_{1}^{\prime }$ = 0. $\alpha_{s}^{\prime },$*s *= 1,..., *K*, are the subpopulation-specific parameters representing the potential heterogeneity of disease risk across subpopulations. In this model, log-odds-ratio *β *measures the association between the genotype and risk of disease, log-odds-ratio *γ *measures the association between the environmental exposure (or another genotype) and risk of disease. The multiplicative interaction *δ *measures the change of the disease-genotype log-odds-ratios according to different levels of risk factor *H*. Similar risk models for studying genetic effect under PS can be found in Satten et al. [21] and Cheng and Lin [17], for examples. For subpopulation *s*, we use *OR_s _*to represent the baseline *G*-*H *odds ratio (given *D *= 0). Define

$$G_{s}=\frac{P\left(G=1|S=s,D=0,H=0\right)}{P\left(G=0|S=s,D=0,H=0\right)}$$

as the baseline *G*- frequency odds and baseline *H*- frequency odds *H_s _*is similarly defined. Also define *D_s _*as the baseline disease frequency odds given by

$$D_{s}=\frac{P\left(D=1|S=s,G=0,H=0\right)}{P\left(D=0|S=s,G=0,H=0\right)}.$$

In the discussion of PS effect, one often assumes that case and control data are sampled according to the SRS design. Let *P*(*S = s|D = *1) and *P*(*S = s|D = *0) represent the corresponding proportions of subpopulation *s *in the cases and controls, respectively. However, in real applications, selection bias often happens and sampling may not be done according to the SRS scheme for various reasons. Let the true proportion of subjects in the cases (controls) that are from subpopulation s be denoted by *P*^#^(*S = s|D = *1) (*P*^#^(*S = s|D = *0)). We use *DS_s _*= $\frac{P^{\text{\# }}\left(S=s|D=1\right)}{P\left(S=s|D=1\right)}/\frac{P^{\text{\# }}\left(S=s|D=0\right)}{P\left(S=s|D=0\right)}$ to measure the effect of the sample selection for subpopulation *s*. If there is no selection bias, *DS_s _*= 1.

Since in the population level we only observe factors *G *and *H*, we show in the Methods section that given the presence of PS and general sampling, the main effects and interaction are given by

$$\begin{array}{c} D-G\;\text{odds}\;\text{ratio}=\text{exp}\left(\beta +\beta^{*}\right),\\ D-H\;\text{odds}\;\text{ratio}=\text{exp}\left(\gamma +\gamma^{*}\right),\\ G\times H\;\text{interaction = exp}\left(\delta +\delta^{*}\right), \end{array}$$

where

$$\begin{array}{c} \beta^{*}=\text{log}\left\{\frac{K\left(1,0\right)}{K\left(0,0\right)}\right\},\\ \gamma^{*}=\text{log}\left\{\frac{K\left(0,1\right)}{K\left(0,0\right)}\right\},\\ \delta^{*}=\text{log}\left\{\frac{K\left(1,1\right)K\left(0,0\right)}{K\left(1,0\right)K\left(0,1\right)}\right\}, \end{array}$$

and

$$\begin{array}{c} K\left(g,h\right)=\\ {}[\overset{K}{\underset{s=1}{\sum }}\left\{P\left(G=0,H=0|S=s,D=0\right)\right\}\times \\ \left\{P^{\#}\left(S=s|D=0\right)OR_{s}^{g\times h}G_{s}^{g}H_{s}^{h}D_{s}DS_{s}\right\}]÷\\ {}[\overset{K}{\underset{s=1}{\sum }}\left\{P\left(G=0,H=0|S=s,D=0\right)\right\}\times \\ \left\{P^{\#}\left(S=s|D=0\right)OR_{s}^{g\times h}G_{s}^{g}H_{s}^{h}\right\}]. \end{array}$$

exp(β*), exp(γ*) and exp(δ*) are the bias levels. We note that if *D_s_DS_s _*is a constant with respect to s, then *K*(*g*, *h*)is also a constant and there is no bias of any kind. A sufficient condition for this to hold is when the baseline disease risk is identical across all subpopulations and sampling of the study follows a SRS design. Further, since

$$\begin{array}{ccc} D_{s}DS_{s} & =\frac{P^{\text{\# }}\left(S=s|D=1\right)}{P^{\text{\# }}\left(S=s|D=0\right)}\times \frac{P\left(D=0|S=s\right)}{P\left(D=1|S=s\right)}\times & \\ & \frac{P\left(D=1|G=H=0,S=s\right)}{P\left(D=0|G=H=0,S=s\right)}\times \frac{P\left(D=1\right)}{P\left(D=0\right)}, \end{array}$$

therefore, if the disease prevalence *P*(*D = *1*|S = s*) and baseline disease risk *P*(*D = *1*|G = H = *0,*S = s*) are approximately equal in each subpopulation, then bias depends on *D_s_DS_s _*only through the degree of matching $\frac{P^{\text{\# }}\left(S=s|D=1\right)}{P^{\text{\# }}\left(S=s|D=0\right)}$. Accordingly, if the case and control are matched in ethnicity, then the bias should be very small. However, *P*(*D = *1*|S = s*) ≈ *P*(*D = *1*|G = H = *0,*S = s*) for all subpopulations is often not true when environmental factor, such as smoking, are involved in causing the disease risk. Under this scenario, even the cases and controls are perfectly matched, the bias can still be large. This conclusion is different from that under the gene-disease association study; see for example, Cheng, Lee and Chen [22]. We shall see more discussion of this issue in latter sections.

### Maximal bias and conditions for the null bias

Here, we give conditions for the null bias and bounds for bias. The bias exp(β*) to the estimation of genetic main effect depends on the variation of the genotype frequencies measured by$G^{†}=\underset{s}{\text{max}}G_{s}/\underset{s}{\text{min}}G_{s},$variation of the disease prevalence measured by $D^{†}=\underset{s}{\text{max}}D_{s}/\underset{s}{\text{min}}D_{s}$and the sampling variation measured by $DS^{†}=\underset{s}{\text{max}}DS_{s}/\underset{s}{\text{min}}DS_{s}.$ The bias exp(δ*) to the estimation of interaction depends additionally on the variation of the baseline odds ratio, measured by $OR^{†}=\underset{s}{\text{max}}OR_{s}/\underset{s}{\text{min}}OR_{s}$ and the variation of exposure rates measured by $H^{†}\text{ = }\underset{s}{\text{max}H_{s}}/\underset{s}{\text{min}}H_{s}.$

Note that the bias β* depends only on *K*(*g*, 0). We first present some conditions for the null bias β* = 0, when the true genetic main effect is null: (1) if the baseline genotype frequency is constant across subpopulations, then the bias β* is zero (can be proved using equation (1) in the Methods section); (2) if the sample selection follows a SRS scheme (*DS*^† ^= 1), and the disease risk is constant, then the bias is also null. (However, if the sampling is not SRS, the bias may be non-null; see Tables 1 and 2.); (3) if the case and control data are matched in ethnicity, and *γ = δ = *0 (both *H*-main effect and interaction are null), then the bias is null.

**Table 1:** Biases and the true type I errors of the chi-square tests when *G*^† ^= 5 and LD = (0,0)

			Bias (*γ *= 0)		type I error (*γ *= 0)		Bias (*γ *= 1)		type I error (*γ *= 1)	
*H*^† ^	*D*^† ^	*DS*^† ^	|β* |	|δ* |	*α_β_*	*α_δ_*	|β* |	|δ* |	*α_β_*	*α_δ_*
1	1	1	0.0000	0.0000	0.0500	0.0500	0.0000	0.0000	0.0500	0.0500
		3	0.2365	0.0000	0.3815	0.0500	0.2365	0.0000	0.3412	0.0500
		5	0.2975	0.0000	0.5513	0.0500	0.2975	0.0000	0.4970	0.0500
		PM	0.0000	0.0000	0.0500	0.0500	0.0000	0.0000	0.0500	0.0500
	3	1	0.3725	0.0000	0.7134	0.0500	0.3725	0.0000	0.6530	0.0500
		3	0.5953	0.0000	0.9823	0.0500	0.5953	0.0000	0.9661	0.0500
		5	0.6518	0.0000	0.9937	0.0500	0.6518	0.0000	0.9857	0.0500
		PM	0.0000	0.0000	0.0500	0.0500	0.0000	0.0000	0.0500	0.0500
	5	1	0.5573	0.0000	0.9602	0.0500	0.5573	0.0000	0.9326	0.0500
		3	0.7679	0.0000	0.9993	0.0500	0.7679	0.0000	0.9977	0.0500
		5	0.8205	0.0000	0.9998	0.0500	0.8205	0.0000	0.9992	0.0500
		PM	0.0000	0.0000	0.0500	0.0500	0.0000	0.0000	0.0500	0.0500
3	1	1	0.0000	0.0000	0.0500	0.0500	0.0000	0.0000	0.0500	0.0500
		3	0.1916	0.1548	0.2583	0.0796	0.1916	0.1548	0.2232	0.0830
		5	0.2383	0.2157	0.3729	0.1074	0.2383	0.2157	0.3201	0.1139
		PM	0.0000	0.0000	0.0500	0.0500	0.0660	0.0285	0.0688	0.0511
	3	1	0.3342	0.0762	0.5794	0.0572	0.3310	0.0796	0.4827	0.0584
		3	0.5134	0.2312	0.9209	0.1163	0.5071	0.2345	0.8439	0.1232
		5	0.5564	0.2892	0.9559	0.1538	0.5493	0.2918	0.8971	0.1632
		PM	0.0000	0.0000	0.0500	0.0500	0.0930	0.0073	0.0812	0.0501
	5	1	0.5129	0.0683	0.8997	0.0557	0.5058	0.0776	0.8083	0.0577
		3	0.6812	0.2225	0.9918	0.1104	0.6687	0.2311	0.9657	0.1187
		5	0.7210	0.2779	0.9962	0.1442	0.7071	0.2852	0.9796	0.1546
		PM	0.0000	0.0000	0.0500	0.0500	0.0957	0.0222	0.0799	0.0506
5	1	1	0.0000	0.0000	0.0500	0.0500	0.0000	0.0000	0.0500	0.0500
		3	0.1608	0.2158	0.1912	0.1113	0.1608	0.2158	0.1639	0.1164
		5	0.1986	0.3042	0.2693	0.1720	0.1986	0.3042	0.2270	0.1816
		PM	0.0000	0.0000	0.0500	0.0500	0.0884	0.0532	0.0815	0.0541
	3	1	0.3005	0.1007	0.4697	0.0635	0.2951	0.1081	0.3676	0.0659
		3	0.4501	0.3178	0.8213	0.1855	0.4405	0.3252	0.6897	0.1942
		5	0.4848	0.4026	0.8762	0.2656	0.4741	0.4085	0.7551	0.2750
		PM	0.0000	0.0000	0.0500	0.0500	0.1325	0.0192	0.1063	0.0505
	5	1	0.4702	0.0892	0.8176	0.0605	0.4574	0.1089	0.6735	0.0655
		3	0.6101	0.3062	0.9661	0.1738	0.5901	0.3249	0.8820	0.1880
		5	0.6423	0.3875	0.9794	0.2470	0.6203	0.4034	0.9122	0.2609
		PM	0.0000	0.0000	0.0500	0.0500	0.1409	0.0474	0.1064	0.0529

PM means that perfect matching *P*^#^(*S *= *s*|*D *= 1) = *P*^#^(*S *= *s*|*D *= 0) is satisfied.

**Table 2:** Biases and true type I errors of the chi-square tests when *G*^† ^= 5 and LD = (0,0.05)

			Bias (*γ *= 0)		type I error (*γ *= 0)		Bias (*γ *= 1)		type I error (*γ *= 1)	
*H*^† ^	*D*^† ^	*DS*^† ^	|β* |	|δ* |	*α_β_*	*α_δ_*	|β* |	|δ* |	*α_β_*	*α_δ_*
1	1	1	0.0000	0.0000	0.0500	0.0500	0.0000	0.0000	0.0500	0.0500
		3	0.1862	0.3173	0.2456	0.1731	0.1862	0.3173	0.2116	0.1886
		5	0.2313	0.4242	0.3535	0.2709	0.2313	0.4242	0.3021	0.2976
		PM	0.0000	0.0000	0.0500	0.0500	0.0710	0.0871	0.0715	0.0598
	3	1	0.3288	0.3309	0.5611	0.1735	0.3281	0.3208	0.4722	0.1791
		3	0.5028	0.6401	0.9076	0.5019	0.5014	0.6166	0.8324	0.5127
		5	0.5443	0.7413	0.9463	0.6209	0.5427	0.7122	0.8873	0.6299
		PM	0.0000	0.0000	0.0500	0.0500	0.0972	0.0634	0.0837	0.0543
	5	1	0.5062	0.4591	0.8883	0.2776	0.5046	0.4356	0.8052	0.2784
		3	0.6695	0.7603	0.9894	0.6206	0.6667	0.7132	0.9643	0.6110
		5	0.7080	0.8563	0.9948	0.7207	0.7048	0.8001	0.9787	0.7072
		PM	0.0000	0.0000	0.0500	0.0500	0.0971	0.0486	0.0806	0.0523
3	1	1	0.0000	0.0000	0.0500	0.0500	0.0000	0.0000	0.0500	0.0500
		3	0.1365	0.4049	0.1484	0.2659	0.1365	0.4049	0.1278	0.2821
		5	0.1677	0.5542	0.2022	0.4417	0.1677	0.5542	0.1700	0.4669
		PM	0.0000	0.0000	0.0500	0.0500	0.0961	0.1592	0.0851	0.0842
	3	1	0.2693	0.3457	0.3779	0.1993	0.2634	0.3503	0.2862	0.2072
		3	0.3958	0.7451	0.6991	0.6654	0.3859	0.7440	0.5461	0.6719
		5	0.4244	0.8876	0.7629	0.8067	0.4135	0.8823	0.6072	0.8083
		PM	0.0000	0.0000	0.0500	0.0500	0.1517	0.0739	0.1175	0.0561
	5	1	0.4286	0.4464	0.7192	0.2912	0.4138	0.4620	0.5509	0.3090
		3	0.5465	0.8394	0.9110	0.7501	0.5248	0.8442	0.7650	0.7536
		5	0.5730	0.9756	0.9361	0.8607	0.5495	0.9731	0.8041	0.8575
		PM	0.0000	0.0000	0.0500	0.0500	0.1656	0.0311	0.1203	0.0510
5	1	1	0.0000	0.0000	0.0500	0.0500	0.0000	0.0000	0.0500	0.0500
		3	0.1034	0.4594	0.1039	0.3341	0.1034	0.4594	0.0917	0.3479
		5	0.1262	0.6322	0.1325	0.5520	0.1262	0.6322	0.1135	0.5712
		PM	0.0000	0.0000	0.0500	0.0500	0.0942	0.2098	0.0812	0.1101
	3	1	0.2198	0.3865	0.2562	0.2424	0.2106	0.4008	0.1850	0.2529
		3	0.3151	0.8406	0.4848	0.7777	0.3007	0.8531	0.3371	0.7791
		5	0.3360	1.0059	0.5407	0.8992	0.3203	1.0147	0.3769	0.8962
		PM	0.0000	0.0000	0.0500	0.0500	0.1623	0.0942	0.1176	0.0597
	5	1	0.3590	0.4966	0.5345	0.3548	0.3343	0.5395	0.3535	0.3893
		3	0.4474	0.9442	0.7431	0.8503	0.4139	0.9825	0.5114	0.8572
		5	0.4667	1.1027	0.7822	0.9352	0.4310	1.1341	0.5467	0.9344
		PM	0.0000	0.0000	0.0500	0.0500	0.1859	0.0365	0.1256	0.0513

PM means that perfect matching *P*^#^(*S *= *s*|*D *= 1) = *P*^#^(*S *= *s*|*D *= 0) is satisfied.

When the interaction effect is null, some conditions for the null bias δ* = 0 are: (1) if the baseline *G*-*H *odds ratios and *G*(or *H*)- frequency odds are constant across subpopulations, then the bias δ* is null (can be proved using equation (2) in the Methods section); (2) if the sample selection of the study follows SRS, and the disease risk is constant, then the bias δ* is also null. However, see Tables 1 and 2 for the presence of bias when the SRS condition fails.

Next, we present bound to measure the largest bias to the estimation of main effect. In the Methods section, we show that the bias exp(β*) can be expressed as

(1) $$\begin{array}{c} \text{exp}\left(\beta^{*}\right)=\frac{\overset{K}{\underset{s=1}{\sum }}G_{s}\left\{D_{s}DS_{s}\right\}w_{s}}{\overset{K}{\underset{s=1}{\sum }}G_{s}w_{s}\overset{K}{\underset{s=1}{\sum }}\left\{D_{s}DS_{s}\right\}w_{s}}\\ \leq \frac{\sqrt{G^{†}D^{†}DS^{†}}{\left(\sqrt{G^{†}D^{†}DS^{†}}+1\right)}^{2}}{\left(\sqrt{G^{†}D^{†}DS^{†}}+G^{†}\right)\left(\sqrt{G^{†}D^{†}DS^{†}}+D^{†}DS^{†}\right)}\\ \equiv U_{\beta }, \end{array}$$

where *w_s _*are some constants satisfying 0 ≤ *w_s _*≤ 1 and $\overset{K}{\underset{s=1}{\sum }}w_{s}=1$. The bias is the greatest when the number of subpopulations is 2. The bias is also bounded below by$L_{\beta }\equiv U_{\beta }^{-1}$. These bounds give the maximal impact of the bias in making inference about the genetic main effect. Under rare disease, the background disease rate is approximately equal to the background disease odds. We find that the bound under SRS (*DS*^† ^= 1) is similar to that given by Lee and Wang [19]. However, our result is more general in the sense that their risk model was a special case of ours and selection bias was not considered in their paper either.

In the Methods section, we also showed that under SRS, the bias exp(δ*) was bounded above by $U_{\delta }^{\left(1\right)}=$(*D*^† ^)^2 ^and bounded below$L_{\delta }^{\left(1\right)}=$(*D*^† ^)^-2^. These are the same bounds derived by Wang et al. [18]. Unfortunately, these bounds are not valid when there is selection bias. Under the general sample selection, we showed that the bias exp(δ*) was bounded above by

(2) $$\begin{array}{ccc} OR^{†} & \times \frac{{\left(\sqrt{G^{†}H^{†}}+1\right)}^{3}}{\left(\sqrt{G^{†}H^{†}}+G^{†}\right)\left(\sqrt{G^{†}H^{†}}+H^{†}\right)}\times & \\ & \;\;\;\;\frac{\left(\sqrt{G^{†}H^{†}}+G^{†}H^{†}\right)}{{\left(\sqrt{G^{†}}+\sqrt{H^{†}}\right)}^{2}}\equiv U_{\delta }^{\left(2\right)}, \end{array}$$

and bounded below by $1/U_{\delta }^{\left(2\right)}\equiv L_{\delta }^{\left(2\right)}$. Using these bounds we can easily conclude that if the genetic factors are in linkage equilibrium within each subpopulation, and the variation of the *G *(or *H*) frequency odds is small then the bias is also expected to be small.

### True type I errors

In case-control studies, one often expects that the type I errors of the association tests can be approximately controlled at some predetermined level. However, in the presence of PS or selection bias, the usual test statistic does not have a chi-square distribution under the null hypothesis. Instead, it has a non-central chi-square distribution, with non-centrality parameter depending on the level of the bias. Thus, the usual chi-square test tends to have inflated type I errors.

Suppose that the intended type I error rate of the chi-square test is *α *and let $\chi_{1;1-\alpha }^{2}$represent the 100(1-*α*) percentile of the chi-square distribution with one degree of freedom. Let $\chi_{1}^{2}\left(\Delta \right)$ represent a non-central chi-square random variable with one degree of freedom and non-centrality parameter Δ. In the case of testing null interaction, the non-centrality parameter is given by

$$\begin{array}{ccc} \Delta_{\delta }= & {\left\{\delta^{*}\right\}}^{2}÷\left\{\left(\frac{1}{n_{11}^{\left(1\right)}}+\frac{1}{n_{01}^{\left(1\right)}}+\frac{1}{n_{10}^{\left(1\right)}}+\frac{1}{n_{00}^{\left(1\right)}}\right)\right)+ & \\ & \left(+\left(\frac{1}{n_{11}^{\left(0\right)}}+\frac{1}{n_{01}^{\left(0\right)}}+\frac{1}{n_{10}^{\left(0\right)}}+\frac{1}{n_{00}^{\left(0\right)}}\right)\right\}, \end{array}$$

where $n_{gh}^{\left(d\right)}$ is number of observations with outcome *G = g*, *H = h *and disease status *d*. Then the true type I error of the usual chi-square test of null interaction is given by $\alpha_{\delta }=P\left(\chi_{1}^{2}\left(\Delta_{\delta }\right)\geq \chi_{1;1-\alpha }^{2}\right),$ which is always ≥ *α*. In the case of testing null genetic main effect, the non-centrality parameter is given by

$$\Delta_{\beta }=\frac{{\left\{\beta^{*}\right\}}^{2}}{\left(\frac{1}{n_{10}^{\left(1\right)}}+\frac{1}{n_{00}^{\left(1\right)}}+\frac{1}{n_{10}^{\left(0\right)}}+\frac{1}{n_{00}^{\left(0\right)}}\right)}.$$

The corresponding true type I error of the chi-square test is given by $\alpha_{\beta }=P\left(\chi_{1}^{2}\left(\Delta_{\beta }\right)\geq \chi_{1;1-\alpha }^{2}\right),$ which is also ≥ *α*.

### Conservative p-values

In most practical applications, one often does not know the true value of the non-centrality parameter and therefore it is difficult to calculate the true p-value of the chi-square test when the PS is present and/or there is selection bias. However, we are able to develop a bound for the non-centrality parameter, and the latter may be estimable in many cases. Define $\Delta_{\delta }^{*}$ ($\Delta_{\beta }^{*}$) as Δ*_δ_*(Δ*_β_*) but with δ* (β*) replaced by its upper bound $\text{log}U_{\delta }^{\left(2\right)}$ (log*U_β_*). Let $\chi_{\delta }^{2}$ ($\chi_{\beta }^{2}$) be the usual statistic for testing null interaction (main effect). Then following Cheng, Lee and Chen [22], a conservative p-value of the chi-square test is given by $P\left(\chi_{1}^{2}\left(\Delta_{\delta }^{*}\right)\geq \chi_{\delta }^{2}\right)$ ($P\left(\chi_{1}^{2}\left(\Delta_{\beta }^{*}\right)\geq \chi_{\beta }^{2}\right)$). We note that by using the property of non-central chi-square distribution, the test based on using conservative p-value always have true type I error rate smaller than or equal to the significance level and the latter is always smaller than or equal to the true type I error rate of the usual chi-square test. If a test has conservative p-value less than or equal to the designated significance level, it is significant even there is PS or selection bias.

### Examples of true biases and type I error rates

Tables 1 and 2 show some values of the biases β* and δ* and true type I error rates *α_β _*and *α_δ _*of the usual chi-square tests when the significance level is 0.05. We assumed that there are two subpopulations (*K *= 2), *β = δ = *0, *γ = *0 or 1. *G *(*H*-) frequency of the first subpopulation was given by *P*(*G *= 1*|S *= 1) = 0.51 (*P*(*H *= 1*|S *= 1) = 0.19), the first subpopulation disease risk was *P*(*D *= 1*|S *= 1) = 0.05, the proportion of subpopulation 1 in the overall population was 0.7, and case and control sample sizes both equaled to *n *= 500. We defined *LD*_s _= (*LD*_1_, *LD*_2_) where *LD*_s _was the linkage disequilibrium coefficient between loci *G *and *H *in subpopulation *s*, and considered linkage disequilibrium coefficient *LD*_s _= 0 or 0.05. We also assumed that the sampling proportions of the cases followed SRS but those of the controls might not. The rest of the parameter values were determined from the values for the variations *G*^† ^,*H*^† ^,*D*^† ^and *DS*^† ^given in the tables with the assumption that subpopulation 2 has the maximal baseline *G *(or *H*) frequency odds, disease risk, and sampling deviation (this implies that *P*^#^(*S *= 2*|D *= 0) ranges from 0.0585 to o.7163). Finally, we note that in computing the non-centrality parameters, the sample frequencies $n_{gh}^{d}$ were replaced by *n *× *P*(*G = g*, *H = h|D = d*). The simulation results for *G*^† ^= 5 were given in Tables 1 and 2, and those for *G*^† ^= 3 can be found from Tables S1 and S2 in Additional file 1.

According to the results in Table 1 the true type I error *α_β _*ranges from 0.05 to 0.9998 under linkage equilibrium. If the SRS condition holds and *γ = *0, the true type I error *α_β _*ranges from 0.05 to 0.9602 with mean 0.4377 and standard error 0.3298. Under the same conditions but *γ = *1, the corresponding range becomes (0.05, 0.9326) with mean 0.3822 and standard error 0.2969. On the other hand, if the sampling is not SRS (*DS*^† ^= 3 or 5) and *γ = *0, the range of *α_β _*is (0.05, 0.9998) with mean 0.6871 and standard error 0.317. Under non-SRS but *γ = *1, the corresponding range becomes (0.05, 0.9992) with mean 0.6291 and standard error 0.3117. These results indicate that the bias can be quite large and its level may be modified by the sample selection and the level of *H*-main effect. We also observe that the bias β* may be nonzero under perfect matching. For example, if matching is perfect and *H*-main effect *γ = *1, the largest true type I error is 0.1064, which occurs at the case with *G*^† ^= *H*^† ^*= D*^† ^= 5. This is contrary to our usual belief that matching between cases and controls in ethnicity can eliminate the PS bias. However, except in some special cases, the bias under perfect matching design are smaller than those under other sampling designs.

Wang et al. [18] suggested that the bias δ* to the interaction effect is small when the linkage disequilibrium coefficient is small and the sampling is SRS. Our Table 1 also shows that under the same condition, the true type I error *α_δ _*in testing null interaction ranges from 0.05 to 0.0659. This agrees with their finding. However, if there is selection bias (*DS*^† ^= 3 or 5), the true type I error rate *α_δ _*has range (0.05, 0.2656), mean 0.101, and standard error 0.056 when *γ = *0, and range (0.05, 0.2750), mean 0.1053, and standard error 0.0597 when *γ = *1. The means and standard errors given here and later were computed based on the results shown in Tables 1 and 2, and Tables S1 and S2 in Additional file 1. These results indicate that PS and SS also can cause serious bias problem in case-control study of gene-gene interactions even when the two genes are in linkage equilibrium. Under this scenario, the best way of reducing the bias is to match cases and controls in ethnicity. We note that under perfect matching and linkage equilibrium, the range of *α_δ _*is only between 0.05, and 0.0541.

Linkage disequilibrium between two genes or correlation between genetic and environmental factors play important role in determining the bias level in the studies of interaction. According to results presented in Table 2 we find that the bias to the estimation of the genetic main effect becomes smaller when the linkage disequilibrium coefficient increases from 0 to 0.05. When *γ = *0, the mean of *α_β _*is 0.3377 under SRS and 0.5514 under non-SRS (selection bias), and when *γ = *1 the mean becomes 0.2716 and 0.4597, under SRS and non-SRS, respectively. On the contrary, the bias to the estimation of the interaction effect increases when the linkage disequilibrium coefficient increases from 0 to 0.05. Our results show that when *γ = *0, the mean of *α_δ _*is 0.1642 under SRS and 0.5512 under non-SRS. When *γ = *1, the mean becomes 0.1706 and 0.5555, under SRS and non-SRS, respectively. In all, bias δ* seems to become larger when linkage disequilibrium coefficient gets larger. Under stronger linkage disequilibrium, the true type I error *α_δ _*can be as large as 0.1101 even the cases and control were perfectly matched.

### An application

Shi et al. [23] studied the interaction effects of maternal smoking and maternal or fetal pharmacogenetic variants on the risk of orofacial cleft based on 1244 subjects from Demark and Iowa, USA with facial clefting and 4183 parents, siblings or unrelated population controls. We considered the combined Denmark and Iowa case-control data with *H *= 1if maternal smoking was yes (0 if no) and *G *= 1if GSTT1 genotype was null (0, if genotype was not-null); see Table A6 of [23]. Based on these data, we found that *G *× *H *interaction was 3.2499 and chi-square test had p-value equal to 5.5676 × 10^-4^, indicating strong interaction effect. Also, from [24] we found that GSTT1 genotype frequencies of the Caucasian populations were between 0.129 and 0.276, giving the variation of the genotype frequencies *G*^† ^= 4.8762. The range of maternal smoking rate was between 0.101 and 0.244 (see [25-27]), giving the variation of exposure rates *H*^† ^= 1.968. Since maternal smoking and GSTT1 were independent in the unrelated control population (p-values of the independence test for the Demark data and Iowa data were respectively equal to 0.0942 and 0.0976), our upper bound for the bias exp(δ*) (see equation 2) equals to 1.6149, leading to the conservative p-value equal to 2.0353 × 10^-2^. This suggests that the maternal smoking effect on the cleft risk can be modified by the GSTT1 genotype even the population stratification and selection bias are both present in the study.

## Discussion

The impact of population stratification is considered by many to be important in case-control studies of gene-disease association. Many authors have suggested quantitative methods to control type I errors of the usual association test. The most popular treatments include the "genomic control" method [28-33] and the "structured association" method [34-37]. Each of the proposed methods requires typing extra polymorphic markers to generate an estimate of PS which can be used to adjust the test statistic. The impact of PS in case-control studies of gene-gene (environment) interaction is considered to be less important, when the genes under studied are in linkage equilibrium or when the gene-environment correlation is weak [18,38]. However, this conclusion holds only when the sampling of the case and control data follow a SRS design, that is no selection bias. Unfortunately, there is no formal method for testing the validity of the SRS condition when the PS is present.

In practical applications, the selection bias is not unusual. For examples, when the hospital-based cases (controls) are used in the study and they are not representative of the population-based cases (controls) or when many non-response of the cases or/and controls occur in the study or there are self-selections, then the SRS condition may fail. In this paper, we show that under slight selection bias (*DS*^† ^= 3), the bias to the estimation of main or interaction effect may become unacceptable. Our suggestion is that the bias should be treated seriously, even when the genetic factors are in linkage equilibrium or the genetic and environmental factors are uncorrelated. Large correlation or strong linkage disequilibrium could make the bias become even larger. Also, small variation in disease risk cannot guarantee small bias, unless there is also small selection bias. In applications, it is important to be able to measure the impact of the bias. In this paper, we drive some bounds for the bias. If these bounds are estimable, then they can be used to make conservative inference. We show one real example that a conservative p-value for testing null interaction can be computed and significance conclusion can be reached even there is bias. Genotype frequencies of the SNPs and their LDs are readily available from international HapMap project. Further, disease prevalence is also available from many nations or from World Health Organization, for example. This information allows us to easily compute bounds and then conservative p-values.

We note that matching in ethnicity between cases and controls has been suggested by epidemiologists as an affective method to control the PS bias in case-control gene-disease association study. However, in a more complicated risk model such as the one discussed here, bias (β*) (see equation 1) to the genetic main effect also depends on the effect size of other risk factor. We found that if *γ = δ = *0 then the residual bias after matching is small. However, if *γ = *1, and *δ = *0, the residual bias after matching is still quite substantial. A sufficient condition to assure bias β* = 0 under perfect matching is *γ = δ = *0. Tables 1 and 2 also show that matching cannot remove bias to the estimation of the interaction effect.

Since the presence of PS and selection bias may cause unacceptable bias to the usual interaction analysis, it is of importance to have an efficient method to control the bias. Unfortunately, so far there exists no effective method. The major difficulty is that the level of the bias depends on the effect size of other related factor which is in general unknown or not estimable under the PS. However, under some special cases, for example, when the genetic main effects are null (or weak) and testing gene-gene interaction is the main focus, one may follow the idea of genomic control to type extra pairs of null markers and apply the computed interaction levels to control the bias. In principle, if the candidate markers are in linkage equilibrium, the selected pairs of null markers also need to be in linkage equilibrium so that the important characteristics of the bias can be captured. On the other hand, if the candidate markers are in linkage disequilibrium, the paired null markers also need to be correlated. We are currently working to solve this important problem. Another approach for reducing bias is to match the cases and controls in ethnicity. According to our simulations, we find that under perfect matching and weak linkage disequilibrium, the bias to the estimation of the interaction effect is small. However, more study is needed in order to understand the impact of the residual bias when the matching is not perfect.

## Conclusions

In this paper, the biases to the estimation of genetic main and interaction effects are quantified and their bounds are derived. We find that if there is environmental effect or interaction, the bias to the genetic main effect cannot be ignored even cases and controls were matched in ethnicity. The bias to the estimation of interaction effect also has the same problem. The estimated bound can be used to compute conservative p-value for the association test. The computation of conservative p-value does not require the knowledge on the number of subpopulations involved in the study or the membership of each study subject. In real applications, it is usually not clear that if there is PS or selection bias or both. However, if appropriate information such as the variation of genotype frequencies is known, we always can compute the conservative p-value. If the conservative p-value is smaller than the designated significance level, we can safely claim that the test is significant regardless of the presence of PS/non-SRS.

## Methods

Following the usual Bayesian argument, the disease-risk model implies that

$$\begin{array}{c} \text{Pr}\left(G=g,\;H=h|S=s,\;D=1\right)÷\\ \text{Pr}\left(G=g,\;H=h|S=s,\;D=0\right)\\ =\text{exp}\left(\mu^{\prime }+\alpha_{s}+\beta g+\gamma h+\delta gh\right), \end{array}$$

where $\alpha_{s}=\alpha_{s}^{\prime }+\text{log}\left\{\text{Pr}\left(D=0,\;S=s\right)\text{Pr}\left(D=1,\;S=s\right)\right\}$, *s = 2,..., k*. As a consequence,

$$\begin{array}{c} \text{Pr}\left(G=g,\;H=h|D=1\right)\\ =\text{exp}\left(\mu^{\prime }+\beta g+\gamma h+\delta gh\right)\times \\ \;\overset{k}{\underset{s=1}{\sum }}[\text{Pr}\left(G=g,\;H=h|S=s,\;D=0\right)\times \\ \;\text{P}{\;}^{\#}\left(S=s|D=1\right)\text{exp}\left(\alpha_{s}\right)]. \end{array}$$

On the other hand, the joint frequency distribution of *G *and *H *in the control population is given by

$$\begin{array}{c} \text{Pr}\left(G=g,\;H=h|D=0\right)\\ =\overset{k}{\underset{s=1}{\sum }}\text{Pr}\left(G=g,\;H=h|S=s,\;D=0\right)\times \\ \text{P}{\;}^{\#}\left(S=s|D=0\right). \end{array}$$

Thus their ratio is given by

$$\begin{array}{c} \text{Pr}\left(G=g,\;H=h|S=s,\;D=1\right)÷\\ \text{Pr}\left(G=g,\;H=h|S=s,\;D=0\right)\\ =\text{exp}\left(\mu^{\prime }+\beta g+\gamma h+\delta gh\right)K^{*}\left(g,\;h\right)\\ =\text{exp}\{\left(\mu^{\prime }+\mu^{*}\right)+\left(\beta_{}+\beta_{}^{*}\right)g+\\ \;\;\;\;\left(\gamma +\gamma^{*}\right)h+\left(\delta_{}+\delta_{}^{*}\right)gh\} \end{array}$$

Here, we define μ* = log{*K*^Δ^(0,0)},$\beta_{}^{*}=\text{log}\left\{\frac{K^{▵}\left(1,0\right)}{K^{▵}\left(0,0\right)}\right\}$,$\gamma_{}^{*}=\text{log}\left\{\frac{K^{▵}\left(0,1\right)}{K^{▵}\left(0,0\right)}\right\}$and $\delta_{}^{*}=\text{log}\left\{\frac{K^{▵}\left(1,1\right)K^{▵}\left(0,0\right)}{K^{▵}\left(0,1\right)K^{▵}\left(1,0\right)}\right\}$, where$K^{▵}\left(g,\;h\right)=K\left(g,h\right)\times \frac{P\left(D=0\right)}{P\left(D=1\right)}.$ Note that the above results are derived using the expression of

$$\begin{array}{ccc} \text{exp}\left(\alpha_{s}\right) & =\frac{P\left(G=H=0|D=1,S=s\right)}{P\left(G=H=0|D=0,S=s\right)} & \\ & =\frac{P\left(D=1|G=H=0,S=s\right)}{P\left(D=0|G=H=0,S=s\right)}\times \\ & \;\frac{P\left(S=s|D=0\right)}{P\left(S=s|D=1\right)}\times \frac{P\left(D=0\right)}{P\left(D=1\right)}. \end{array}$$

Also note that we can express

(3) $$\text{exp}\left(\beta^{*}\right)=\frac{\overset{k}{\underset{s=1}{\sum }}G_{s}\left\{D_{s}DS_{s}\right\}w_{s}}{\overset{k}{\underset{s=1}{\sum }}G_{s}w_{s}\overset{k}{\underset{s=1}{\sum }}\left\{D_{s}DS_{s}\right\}w_{s}},$$

where$w_{s}=w_{s}^{*}/\overset{K}{\underset{s=1}{\sum }}w_{s}^{*}$and

$$\begin{array}{ccc} w_{s}^{*}= & P\left(G=0,H=0|S=s,D=0\right)\times & \\ & P^{\text{\# }}\left(S=s|D=0\right) \end{array}$$

Define

$$U_{m}^{M}\left(w\right)=wG_{M}D_{M}DS_{M}+\left(1-w\right)G_{m}D_{m}DS_{m}$$

and

$$V_{M}^{m}\left(w\right)=wG_{M}+\left(1-w\right)G_{m}.$$

Simple algebra shows that there exists some constant w* such that the bias is bounded above by

$$\begin{array}{c} \frac{U_{M}^{m}\left(w^{*}\right)}{U_{M}^{m}\left(w^{*}\right)\times V_{M}^{m}\left(w^{*}\right)}\\ \leq \underset{0\leq w\leq 1}{\text{max}}\frac{U_{M}^{m}\left(w\right)}{U_{M}^{m}\left(w\right)\times V_{M}^{m}\left(w\right)}\\ =\frac{\sqrt{G^{†}D^{†}DS^{†}}{\left(\sqrt{G^{†}D^{†}DS^{†}}+1\right)}^{2}}{\left(\sqrt{G^{†}D^{†}DS^{†}}+G^{†}\right)\left(\sqrt{G^{†}D^{†}DS^{†}}+D^{†}DS^{†}\right)}. \end{array}$$

Here *G_M_*(*G_m_*) is the largest value of *G_s_*.*D_M_*, *D_m_*, *DS_M_*, and *DS_m _*are similarly defined. Also note that under SRS, *DS_s _*= 1 and therefore according to the definition of exp(δ*)we easily show that it is bounded above by (*D*^† ^)^2 ^and bounded below by (*D*^† ^)^-2^. However, under general sampling design, the bias is expressed as

(4) $$\begin{array}{ccc} \text{exp}\left(\delta^{*}\right)= & \frac{\overset{K}{\underset{s=1}{\sum }}OR_{s}G_{s}H_{s}{w_{s}}^{\prime }}{\overset{K}{\underset{s=1}{\sum }}G_{s}{w_{s}}^{\prime }\overset{K}{\underset{s=1}{\sum }}H_{s}{w_{s}}^{\prime }} & \\ & \times \frac{\overset{K}{\underset{s=1}{\sum }}G_{s}{w^{\prime \prime }}_{s}\overset{K}{\underset{s=1}{\sum }}H_{s}{w^{\prime \prime }}_{s}}{\overset{K}{\underset{s=1}{\sum }}OR_{s}G_{s}H_{s}{w^{\prime \prime }}_{s}}, \end{array}$$

where $w_{s}^{\prime }=\frac{D_{s}DS_{s}P^{\text{\# }}\left(S=s|D=0\right)}{\overset{k}{\underset{s^{\prime }=1}{\sum }}D_{s^{\prime }}DS_{s^{\prime }}P^{\text{\# }}\left(S=s^{\prime }|D=0\right)}$and $w_{s}^{\prime \prime }=\frac{P^{\text{\# }}\left(S=s|D=0\right)}{\overset{k}{\underset{s^{\prime }=1}{\sum }}P^{\text{\# }}\left(S=s^{\prime }|D=0\right)}$. By applying the same approach for deriving bounds for exp(β*), we also can derive bounds for exp(δ*).

## Authors' contributions

KFC designed the study, performed the analysis and wrote the paper. JYL performed the Computation and helped in discussion. All authors read and approved the final manuscript.

## Supplementary Material

Additional file 1 **Biases and the true type I errors of the chi-square tests**. The file contains two tables showing the biases and true type I errors of the chi-square tests when *G*^† ^= 3 and LD = (0,0) or LD = (0,0.5). Click here for file
