# Supplementary material for: Assessing the joint effect of population stratification and sample selection in studies of gene-gene (environment) interactions
Source: BMC Genet. 2012 Jan 27;13:5. doi: 10.1186/1471-2156-13-5 (PMC3280159; doi:10.1186/1471-2156-13-5)
Supplement: Additional file 1 — Biases and the true type I errors of the chi-square tests. The file contains two tables showing the biases and true type I errors of the chi-square tests when G† = 3 and LD = (0,0) or LD = (0,0.5). [file 1471-2156-13-5-S1.DOC]

The following tables show true biases and type I error rates of the chi-square test under$G^{\dagger}$=3. Other conditions are given in the main text.

Table S1. Biases and the true type I errors of the chi-square tests when $G^{\dagger}$=3 and LD=(0,0)

|  |  |  | Bias  (*γ*=0) | | type I error  (*γ*=0) | |  | Bias  (*γ*=1) | | type I error  (*γ*=1) | |
| --- | --- | --- | --- | --- | --- | --- | --- | --- | --- | --- | --- |
| $H^{\dagger}$ | $D^{\dagger}$ | $DS^{\dagger}$ |  |  | $\alpha_{\beta}$ | $\alpha_{\delta}$ |  |  |  | $\alpha_{\beta}$ | $\alpha_{\delta}$ |
| 1 | 1 | 1 | 0.0000 | 0.0000 | 0.0500 | 0.0500 |  | 0.0000 | 0.0000 | 0.0500 | 0.0500 |
|  |  | 3 | 0.1761 | 0.0000 | 0.2370 | 0.0500 |  | 0.1761 | 0.0000 | 0.2132 | 0.0500 |
|  |  | 5 | 0.2219 | 0.0000 | 0.3470 | 0.0500 |  | 0.2219 | 0.0000 | 0.3105 | 0.0500 |
|  |  | PM | 0.0000 | 0.0000 | 0.0500 | 0.0500 |  | 0.0000 | 0.0000 | 0.0500 | 0.0500 |
|  | 3 | 1 | 0.2697 | 0.0000 | 0.4627 | 0.0500 |  | 0.2697 | 0.0000 | 0.4143 | 0.0500 |
|  |  | 3 | 0.4358 | 0.0000 | 0.8576 | 0.0500 |  | 0.4358 | 0.0000 | 0.8075 | 0.0500 |
|  |  | 5 | 0.4782 | 0.0000 | 0.9141 | 0.0500 |  | 0.4782 | 0.0000 | 0.8740 | 0.0500 |
|  |  | PM | 0.0000 | 0.0000 | 0.0500 | 0.0500 |  | 0.0000 | 0.0000 | 0.0500 | 0.0500 |
|  | 5 | 1 | 0.3991 | 0.0000 | 0.7818 | 0.0500 |  | 0.3991 | 0.0000 | 0.7241 | 0.0500 |
|  |  | 3 | 0.5563 | 0.0000 | 0.9694 | 0.0500 |  | 0.5563 | 0.0000 | 0.9467 | 0.0500 |
|  |  | 5 | 0.5958 | 0.0000 | 0.9841 | 0.0500 |  | 0.5958 | 0.0000 | 0.9692 | 0.0500 |
|  |  | PM | 0.0000 | 0.0000 | 0.0500 | 0.0500 |  | 0.0000 | 0.0000 | 0.0500 | 0.0500 |
| 3 | 1 | 1 | 0.0000 | 0.0000 | 0.0500 | 0.0500 |  | 0.0000 | 0.0000 | 0.0500 | 0.0500 |
|  |  | 3 | 0.1432 | 0.1099 | 0.1659 | 0.0652 |  | 0.1432 | 0.1099 | 0.1462 | 0.0668 |
|  |  | 5 | 0.1783 | 0.1550 | 0.2328 | 0.0800 |  | 0.1783 | 0.1550 | 0.2017 | 0.0831 |
|  |  | PM | 0.0000 | 0.0000 | 0.0500 | 0.0500 |  | 0.0489 | 0.0181 | 0.0605 | 0.0505 |
|  | 3 | 1 | 0.2449 | 0.0395 | 0.3622 | 0.0521 |  | 0.2428 | 0.0423 | 0.2979 | 0.0525 |
|  |  | 3 | 0.3790 | 0.1504 | 0.7128 | 0.0792 |  | 0.3746 | 0.1533 | 0.6053 | 0.0820 |
|  |  | 5 | 0.4113 | 0.1935 | 0.7839 | 0.0980 |  | 0.4062 | 0.1959 | 0.6776 | 0.1020 |
|  |  | PM | 0.0000 | 0.0000 | 0.0500 | 0.0500 |  | 0.0664 | 0.0100 | 0.0666 | 0.0502 |
|  | 5 | 1 | 0.3725 | 0.0255 | 0.6720 | 0.0509 |  | 0.3679 | 0.0327 | 0.5597 | 0.0515 |
|  |  | 3 | 0.4984 | 0.1365 | 0.9031 | 0.0740 |  | 0.4899 | 0.1436 | 0.8094 | 0.0775 |
|  |  | 5 | 0.5284 | 0.1778 | 0.9339 | 0.0904 |  | 0.5187 | 0.1839 | 0.8528 | 0.0948 |
|  |  | PM | 0.0000 | 0.0000 | 0.0500 | 0.0500 |  | 0.0666 | 0.0199 | 0.0655 | 0.0506 |
| 5 | 1 | 1 | 0.0000 | 0.0000 | 0.0500 | 0.0500 |  | 0.0000 | 0.0000 | 0.0500 | 0.0500 |
|  |  | 3 | 0.1205 | 0.1518 | 0.1284 | 0.0809 |  | 0.1205 | 0.1518 | 0.1133 | 0.0832 |
|  |  | 5 | 0.1489 | 0.2171 | 0.1725 | 0.1128 |  | 0.1489 | 0.2171 | 0.1486 | 0.1172 |
|  |  | PM | 0.0000 | 0.0000 | 0.0500 | 0.0500 |  | 0.0657 | 0.0325 | 0.0675 | 0.0516 |
|  | 3 | 1 | 0.2218 | 0.0510 | 0.2901 | 0.0537 |  | 0.2180 | 0.0569 | 0.2281 | 0.0547 |
|  |  | 3 | 0.3339 | 0.2050 | 0.5787 | 0.1085 |  | 0.3270 | 0.2114 | 0.4537 | 0.1123 |
|  |  | 5 | 0.3600 | 0.2678 | 0.6471 | 0.1490 |  | 0.3522 | 0.2731 | 0.5128 | 0.1529 |
|  |  | PM | 0.0000 | 0.0000 | 0.0500 | 0.0500 |  | 0.0956 | 0.0230 | 0.0802 | 0.0508 |
|  | 5 | 1 | 0.3443 | 0.0317 | 0.5700 | 0.0514 |  | 0.3356 | 0.0468 | 0.4373 | 0.0531 |
|  |  | 3 | 0.4493 | 0.1866 | 0.8094 | 0.0982 |  | 0.4352 | 0.2018 | 0.6566 | 0.1049 |
|  |  | 5 | 0.4734 | 0.2470 | 0.8508 | 0.1338 |  | 0.4579 | 0.2602 | 0.7029 | 0.1405 |
|  |  | PM | 0.0000 | 0.0000 | 0.0500 | 0.0500 |  | 0.0994 | 0.0414 | 0.0795 | 0.0525 |

PM means that perfect matching$P^{\text{\#}}(S=s|D=1)=P^{\text{\#}}(S=s|D=0)$is satisfied.

Table S2. Biases and true type I errors of the chi-square tests when $G^{\dagger}$=3 and LD=(0,0.05)

|  |  |  | Bias  (*γ*=0) | | type I error  (*γ*=0) | |  | Bias  (*γ*=1) | | type I error  (*γ*=1) | |
| --- | --- | --- | --- | --- | --- | --- | --- | --- | --- | --- | --- |
| $H^{\dagger}$ | $D^{\dagger}$ | $DS^{\dagger}$ |  |  | $\alpha_{\beta}$ | $\alpha_{\delta}$ |  |  |  | $\alpha_{\beta}$ | $\alpha_{\delta}$ |
| 1 | 1 | 1 | 0.0000 | 0.0000 | 0.0500 | 0.0500 |  | 0.0000 | 0.0000 | 0.0500 | 0.0500 |
|  |  | 3 | 0.1476 | 0.2758 | 0.1741 | 0.1427 |  | 0.1476 | 0.2758 | 0.1536 | 0.1551 |
|  |  | 5 | 0.1841 | 0.3656 | 0.2461 | 0.2140 |  | 0.1841 | 0.3656 | 0.2138 | 0.2354 |
|  |  | PM | 0.0000 | 0.0000 | 0.0500 | 0.0500 |  | 0.0443 | 0.0636 | 0.0587 | 0.0554 |
|  | 3 | 1 | 0.2488 | 0.2993 | 0.3760 | 0.1553 |  | 0.2485 | 0.2888 | 0.3179 | 0.1595 |
|  |  | 3 | 0.3871 | 0.5671 | 0.7358 | 0.4261 |  | 0.3864 | 0.5437 | 0.6452 | 0.4340 |
|  |  | 5 | 0.4207 | 0.6518 | 0.8060 | 0.5284 |  | 0.4199 | 0.6236 | 0.7189 | 0.5353 |
|  |  | PM | 0.0000 | 0.0000 | 0.0500 | 0.0500 |  | 0.0567 | 0.0478 | 0.0626 | 0.0527 |
|  | 5 | 1 | 0.3769 | 0.4170 | 0.6891 | 0.2510 |  | 0.3763 | 0.3922 | 0.5976 | 0.2483 |
|  |  | 3 | 0.5070 | 0.6768 | 0.9160 | 0.5484 |  | 0.5057 | 0.6299 | 0.8486 | 0.5332 |
|  |  | 5 | 0.5381 | 0.7570 | 0.9444 | 0.6407 |  | 0.5366 | 0.7019 | 0.8886 | 0.6211 |
|  |  | PM | 0.0000 | 0.0000 | 0.0500 | 0.0500 |  | 0.0542 | 0.0368 | 0.0608 | 0.0515 |
| 3 | 1 | 1 | 0.0000 | 0.0000 | 0.0500 | 0.0500 |  | 0.0000 | 0.0000 | 0.0500 | 0.0500 |
|  |  | 3 | 0.1118 | 0.3291 | 0.1165 | 0.1953 |  | 0.1118 | 0.3291 | 0.1032 | 0.2065 |
|  |  | 5 | 0.1379 | 0.4517 | 0.1534 | 0.3209 |  | 0.1379 | 0.4517 | 0.1324 | 0.3400 |
|  |  | PM | 0.0000 | 0.0000 | 0.0500 | 0.0500 |  | 0.0693 | 0.1145 | 0.0689 | 0.0688 |
|  | 3 | 1 | 0.2117 | 0.2633 | 0.2633 | 0.1432 |  | 0.2081 | 0.2660 | 0.2060 | 0.1471 |
|  |  | 3 | 0.3156 | 0.5888 | 0.5234 | 0.4960 |  | 0.3093 | 0.5861 | 0.4026 | 0.4975 |
|  |  | 5 | 0.3395 | 0.7058 | 0.5878 | 0.6401 |  | 0.3324 | 0.6995 | 0.4553 | 0.6372 |
|  |  | PM | 0.0000 | 0.0000 | 0.0500 | 0.0500 |  | 0.1032 | 0.0452 | 0.0836 | 0.0527 |
|  | 5 | 1 | 0.3315 | 0.3341 | 0.5263 | 0.1994 |  | 0.3229 | 0.3433 | 0.3967 | 0.2072 |
|  |  | 3 | 0.4286 | 0.6549 | 0.7608 | 0.5753 |  | 0.4152 | 0.6541 | 0.5985 | 0.5698 |
|  |  | 5 | 0.4506 | 0.7667 | 0.8046 | 0.7045 |  | 0.4361 | 0.7596 | 0.6431 | 0.6914 |
|  |  | PM | 0.0000 | 0.0000 | 0.0500 | 0.0500 |  | 0.1083 | 0.0148 | 0.0833 | 0.0503 |
| 5 | 1 | 1 | 0.0000 | 0.0000 | 0.0500 | 0.0500 |  | 0.0000 | 0.0000 | 0.0500 | 0.0500 |
|  |  | 3 | 0.0879 | 0.3644 | 0.0894 | 0.2351 |  | 0.0879 | 0.3644 | 0.0808 | 0.2441 |
|  |  | 5 | 0.1076 | 0.5053 | 0.1104 | 0.3962 |  | 0.1076 | 0.5053 | 0.0970 | 0.4108 |
|  |  | PM | 0.0000 | 0.0000 | 0.0500 | 0.0500 |  | 0.0722 | 0.1455 | 0.0689 | 0.0811 |
|  | 3 | 1 | 0.1796 | 0.2745 | 0.1923 | 0.1559 |  | 0.1738 | 0.2850 | 0.1464 | 0.1611 |
|  |  | 3 | 0.2608 | 0.6364 | 0.3668 | 0.5733 |  | 0.2515 | 0.6454 | 0.2624 | 0.5706 |
|  |  | 5 | 0.2789 | 0.7714 | 0.4137 | 0.7305 |  | 0.2686 | 0.7772 | 0.2943 | 0.7218 |
|  |  | PM | 0.0000 | 0.0000 | 0.0500 | 0.0500 |  | 0.1176 | 0.0471 | 0.0880 | 0.0529 |
|  | 5 | 1 | 0.2884 | 0.3415 | 0.3939 | 0.2127 |  | 0.2738 | 0.3716 | 0.2704 | 0.2308 |
|  |  | 3 | 0.3640 | 0.6997 | 0.5879 | 0.6463 |  | 0.3432 | 0.7258 | 0.4021 | 0.6479 |
|  |  | 5 | 0.3807 | 0.8292 | 0.6306 | 0.7826 |  | 0.3584 | 0.8495 | 0.4337 | 0.7741 |
|  |  | PM | 0.0000 | 0.0000 | 0.0500 | 0.0500 |  | 0.1299 | 0.0053 | 0.0906 | 0.0500 |

PM means that perfect matching$P^{\text{\#}}(S=s|D=1)=P^{\text{\#}}(S=s|D=0)$is satisfied.
